# Embryonic expression of *endothelins* and their receptors in lamprey and frog reveals stem vertebrate origins of complex Endothelin signaling

**DOI:** 10.1038/srep34282

**Published:** 2016-09-28

**Authors:** Tyler Square, David Jandzik, Maria Cattell, Andrew Hansen, Daniel Meulemans Medeiros

**Affiliations:** 1 Department of Ecology and Evolutionary Biology, University of Colorado, Boulder, CO 80309, USA; 2 Department of Zoology, Faculty of Natural Sciences, Comenius University in Bratislava, Bratislava, 84215, Slovakia

## Abstract

Neural crest cells (NCCs) are highly patterned embryonic cells that migrate along stereotyped routes to give rise to a diverse array of adult tissues and cell types. Modern NCCs are thought to have evolved from migratory neural precursors with limited developmental potential and patterning. How this occurred is poorly understood. Endothelin signaling regulates several aspects of NCC development, including their migration, differentiation, and patterning. In jawed vertebrates, Endothelin signaling involves multiple functionally distinct ligands (Edns) and receptors (Ednrs) expressed in various NCC subpopulations. To test the potential role of endothelin signaling diversification in the evolution of modern, highly patterned NCC, we analyzed the expression of the complete set of endothelin ligands and receptors in the jawless vertebrate, the sea lamprey (*Petromyzon marinus*). To better understand ancestral features of gnathostome *edn* and *ednr* expression, we also analyzed all known Endothelin signaling components in the African clawed frog (*Xenopus laevis*). We found that the sea lamprey has a gnathsotome-like complement of *edn* and *ednr* duplicates, and these genes are expressed in patterns highly reminiscent of their gnathostome counterparts. Our results suggest that the duplication and specialization of vertebrate Endothelin signaling coincided with the appearance of highly patterned and multipotent NCCs in stem vertebrates.

Neural crest cells (NCCs) are multipotent migratory embryonic cells that give rise to an array of adult tissues and cell types, including parts of the heart, peripheral ganglia, pigment cells, and much of the head skeleton^1^. Because NCCs are unique to vertebrates, and form a variety of vertebrate-specific derivatives, the origin and evolution of NCCs is considered a major facilitator of vertebrate diversification and success^2^,^3^,^4^.

The NCCs of all living vertebrates are divided into distinct subpopulations with unique migration routes and developmental fates^5^,^6^. Modern NCCs presumably evolved step-wise from a more homogenous population of migratory neural tube cells with limited developmental potential^7^,^8^. When and how NCCs acquired their multipotency, patterning, and stereotyped migration routes, is unclear.

In modern jawed vertebrates, Endothelins are key regulators of NCC differentiation, migration, and patterning^9^,^10^,^11^,^12^,^13^,^14^,^15^,^16^,^17^,^18^,^19^. Gnathostomes possess multiple (2–6) endothelin receptors (*ednr*s)^20^ as well as multiple (3–6) endothelin ligands (*edn*s)^21^. Phylogenetic analysis places the ligands into four paralogy groups^21^: *edn1*, *edn2*, *edn3*, and *edn4*, while the receptors form three groups^20^: *ednra*, *ednrb1*, and *ednrb2*. Importantly, most modern vertebrates possess subsets of these (discussed below).

Endothelin ligands are translated as ~200 aa (amino acid) polypeptides (Preproendothelins) that are then processed into short, and invariably 21 aa signaling ligands. This occurs as Preproendothelins obtain disulphide linkages between cysteine residues 1 and 15, and 3 and 11 (present on all known Edn ligands), and lose their amino and carboxyl termini via cleavage by a furin endopeptidase, making an intermediate product known as “Big Endothelin”. Big Endothelins are subsequently trimmed on the carboxyl end again by an Endothelin converting enzyme (ECE)^22^,^23^,^24^, leaving the 21 aa polypeptide. In jawed vertebrates there are two main groups of ECEs, ECE–1 and −2, which have unique biochemical properties^25^ as well as unique developmental roles in mouse^26^. Despite these differences, both ECE-1 and ECE-2 have a similar hierarchy of Big Endothelin cleavage rates *in vitro* (Edn1 ≫ Edn2 > Edn3)^25^ (Edn4 has not been assayed in this context). Once fully processed and secreted by the cell, mature Edn ligands interact with the various Ednrs to drive cell fate decisions and provide positional information to NCCs^9^,^10^,^11^,^12^,^13^,^14^,^15^,^16^,^17^,^18^. In cell culture assays, each Ednr appears to have a unique set of binding affinities for the different mature Edn ligands^27^,^28^,^29^, though the early developmental relevance of these differences remains unknown.

The first role described for Endothelin signaling was in vasoconstriction and circulatory development^30^,^31^,^32^,^33^. Subsequently, Endothelin pathways were found to perform a variety of functions in NCC including cell fate determination, migratory pathfinding, and patterning of cranial NCC. Specifically, the Edn1/Ednra pathway works to pattern skeletogenic cranial NCC into distinct skeletal progenitor populations along the dorso-ventral axis of the pharynx, thereby positioning the jaw joint, and also has a function in cardiac NCC development^9^,^14^,^34^,^35^,^36^. In non-skeletogenic NCCs, the Edn3/Ednrb pathway is necessary for pigment cell specification and migration^10^,^13^,^18^,^37^, as well as enteric neuron development^10^,^19^. *edn2*, though present in all major vertebrate lineages, does not have known expression in early development to our knowledge. *edn4* is retained only in some lineages of ray-finned fishes^21^, and its expression and function in early development is unknown. Interestingly, *ednrb2* has been lost in both zebrafish and therian mammals^20^, despite this receptor’s importance in NCC-derived pigment cell development in other vertebrates^13^,^18^,^37^.

When the various roles for endothelin signaling in vertebrate NCC development evolved is unknown. *ednr*s appear to be unique to chordates, and are not found in the genomes of protostomes or non-chordate deuterostomes^20^: no tunicate genome appears to contain any *edn-* or *ednr-*like genes^20^,^21^. Amphioxus (*Branchoistoma floridae*) also lacks any Edn ligands^20^,^21^, though it possesses a single *ednr-like* gene that is not expressed during early embryogenesis (unpublished results, ref. 38). The Japanese lamprey, *Lethenteron japonicum*, a jawless vertebrate, possesses at least one *ednr* and six putative *edn* ligands^39^, suggesting that at least a simple form of Endothelin signaling (i.e. a single receptor with its particular ligand affinities) arose before the common ancestor of jawed and jawless vertebrates.

After exhausting available genomic assemblies and multiple transcript assemblies from adult and larval tissues, here we identify and characterize the expression of what is likely the complete set of *ednr* and *edn* ligands in the sea lamprey, *Petromyzon marinus*. We find dynamic embryonic and larval expression of two *ednr*s which are likely orthologous to gnathostome *ednra* and *ednrb*. We also identify six *P. marinus edn* ligand genes, four of which are expressed in temporo-spatially restricted patterns in embryos and early larvae, with a fifth transcribed diffusely throughout the animal. We then use comparisons with *Xenopus laevis ednr* and *edn* expression to assign tentative functional overlap between some lamprey and gnathostome Endothelin receptors and ligands. Our results suggest that at least one *ednr* duplication occurred prior to the divergence of cyclostomes and gnathostomes, and that sophisticated NCC pattering by duplicated and sub- or neofunctionalized *ednr*s and *edn*s likely evolved in stem vertebrates. We posit that the deployment of Ednr signaling in NCC, followed by duplication and divergence of Edn signaling components, paved the way for fine-tuned control over NCC fate determination, migration, and patterning in modern vertebrates.

## Methods

### Gene cloning and sequence acquisition

Polymerase chain reactions (PCRs) were performed according to standard protocols using the primers listed in Table S1. Fragments corresponding to *P. marinus ednA*, *ednC*, *ednE*, *ednra*, and *ednrb* were amplified from a 5′ RACE library made from combined cDNA. These genes were named based on their deduced orthology to *L. japonicum* sequences (see below) except *ednrb*, which has not been identified in *L. japonicum* to our knowledge. Notably, part of the *ednrb* sequence fragment we cloned via RACE had been previously identified^21^ in the 2007 *P. marinus* genomic assembly on contig56749, though only ~350 nt of this sequence is represented there. Unfortunately, this contig is only ~4.9 kilobases long, and does not contain any other predicted coding sequences that could be used to determine synteny. This sequence is absent from the 2010 *P. marinus* genomic assembly. Using the nucleotide sequences published for the Japanese lamprey^39^ as a reference, fragments corresponding to the putative *P. marinus ednB*, *ednD*, and *ednF* transcripts were amplified from genomic DNA or cDNA by designing primers against sequenced transcripts^40^. In the case of *ednD*, our *P. marinus* gene fragment appears to be completely situated within the 3′ UTR. Similarly, seven *P. marinus* metalloprotease transcript fragments (with sequence similarity to gnathostome ECEs) were identified in transcriptome assemblies derived from adult^40^ and combined adult and larval tissues (unpublished data). All sequences have been deposited to GenBank (see Table S2 for accession numbers).

As is typical within the tetraploid *X. laevis* genome, we found two duplicates of each *edn* and *ednr* gene, except in the case of *ednrb2*, for which there appears to be three copies (-a: NM_001086238.1; -b: NM_001085878.1; -c: KU680750). *edn1-b*, *edn3-a*, *ednra-a*, *ednra-b*, *ednrb2-a, and ednrb2-b* were previously described and/or sequenced^11^,^18^ (see accession numbers in Table S2). The riboprobe template for *X. laevis ednra-b* was a gift from the Mayor lab; the rest of these were subcloned from gDNA or cDNA using available sequence information. Using *X. laevis* genomic assemblies 7.1–8.www.xenbase.org), we identified and cloned fragments of *edn1-a*, *edn2-a*, *edn2-b*, *edn3-b*, *ednrb1-a*, *ednrb1-b*, and *ednrb2-c* out of genomic DNA or cDNA. These novel *edn* and *ednr* sequence fragments have been deposited to GenBank (see Table S2 for accession numbers). ClustalW alignments with translated sequence fragments, and subsequent ML trees confirmed the identity of these *Xenopus* sequences.

### *In situ* hybridizations, cryosectioning, and imaging

Using riboprobes generated from the amplified gene fragments described above, *in situ* hybridization (ISH) was performed as previously described^41^,^42^. Developmental staging for lamprey followed Tahara, 1988, while *X. laevis* staging followed Nieuwkoop and Faber, 1994. For simplicity, we use “st. 33,” “st. 35,” and “st. 37” to describe the *X. laevis* developmental windows usually referred to as “st. 33/34,” “st. 35/36,” and “st. 37/38,” respectively. Interestingly, riboprobes designed against the different *X. laevis*-specific gene duplicates of *ednra* (*ednra-a* and *ednra-b*) and *edn2* (*edn2-a* and *edn2-b*) displayed differential staining despite high sequence similarity and regional overlap of the probe binding sites, as previously noted for other *X. laevis*-specific gene duplicates^42^ (see below). Cryosectioning and imaging was performed as previously described^42^,^43^.

### Phylogenetic reconstruction

To assign the orthology of the *P. marinus edn* ligand fragments described above to those previously published for *L. japonicum edn* genes, a ClustalW nucleotide alignment was constructed using only endothelin ligand DNA sequences derived from both lamprey species. The Maximum Likelihood (ML) method of phylogeny reconstruction was employed in MEGA6 thereafter (Fig. S1). The *P. marinus* genes were named corresponding to their orthology with their closest *L. japonicum* relative, for which all ortholog pairs show strong support in a bootstrap analysis.

In an attempt to address the identity of the lamprey Endothelin signaling components amongst all vertebrate Endothelin ligands (save EdnB and EdnD), Endothelin receptors, and Endothelin converting enzymes, ClustalW protein alignments were built using inferred aa sequences derived from transcript or genomic data, and thereafter used to build ML trees (Figs S2, S3 and S4). For the Edn ligands, an ML tree was first built by using aa sequences derived from a variety of gnathostomes (specifically excluding those that were missing the conserved region containing and surrounding the functional ligand), testing many parameters for the aa alignment and ML tree preliminarily. Given the heterogeneity in sequence conservation across Edn aa sequences, these trees were sometimes structured very differently from each other when different parameters were employed, as reflected by the range of bootstrap values and overall tree topology in Fig. S2. We thus selected parameters that would maximize our tree to reflect the accepted relationships of (1) vertebrate taxa, and (2) the synteny analysis on *edn* genes performed previously^21^ which supported *edn1/3* and *edn2/4* clades. We then added *P. marinus* aa sequences to the dataset, realigned, and rebuilt the tree, bootstrapping it 100 times using these gnathostome-optimized parameters (*P. marinus* EdnB and -D, and *L. japonicum* EdnB, -D, -E, and -F were discounted from this analysis due to their incomplete nature). An Endothelin receptor ML tree was similarly constructed, using amphioxus Ednr-like as an outgroup (Fig. S3). To address the identity of putative *P. marinus ECEs* and other related metallopeptidase^44^,^45^,^46^ genes, a phylogeny was built using the inferred translations from each of seven sea lamprey M13 family metallopeptidase transcripts, along with aa sequences for these genes from an assortment of deuterostomes, using three LTA4H sequences as an outgroup (an M1 family peptidase^44^; Fig. S4).

## Results

### Cloning and sequence analysis of endothelin pathway genes

In order to understand the relative timing of the appearance and diversification of Endothelin pathway gene groups, we gathered available sequences from a wide range of deuterostomes (mainly gnathostomes). No gene with moderate sequence similarity to an Edn has been identified in any invertebrate by our own, or previous analyses^20^,^21^, however *ECE-like1* metalloproteases do appear to exist in amphioxus and urchin (Fig. S4), and an amphioxus *ednr-like* gene has been previously identified^20^. It is important to note that ECE-like1 (also known as X-converting enzyme, XCE) is a distinct group of proteins found throughout deuterostomes, and is closely related to, but separate from the clade of proteins containing gnathostome ECE-1 and ECE-2 (Fig. S4). Conversely, the single *Ednr-like* gene in amphioxus appears to be a true ortholog of vertebrate *Ednr*s^20^; there is no known clade of “*ednr-like*” genes in vertebrates.

The ML trees generated here fail to strongly support strict orthology of lamprey and gnathostome Edn ligands and ECE peptidases (Figs S2 and S4). Interestingly, Sea lamprey EdnA clusters with the tetrapod Edn1 group with moderate bootstrap support (71%), however in this analysis, the ray-finned fish Edn1 sequences were excluded from this group. Conversely, *P. marinus* Ednra and Ednrb cluster separately with their putative gnathostome orthologs, with moderate bootstrap support (59%, and 58% respectively; see Fig. S3). Thus while a parsimonious view would suggest that these two lamprey receptors do belong to these two main groups of Ednrs, it remains possible that one or both of these lamprey Ednrs are actually duplicates that were lost in gnathostomes, which would make them a unique Ednr subtype. Sea lamprey appear to possess five *ECE* genes, which we have named *ECE-A*, *-B*, *-C*, *-D*, and -*E*. Notably, ECE-A and -B cluster with the gnathostome ECE-1/2 clade with high bootstrap support (see Fig. S4), whereas ECE-C, -D, and -E have low support, and thus these latter three independent duplicates might not actually code for enzymes capable of processing Edns. We have named them as such simply because they show the strongest sequence similarity to ECEs. All accession numbers corresponding to these sequenced transcripts can be found in Table S2.

### Expression of *P. marinus* endothelin signaling components

We assayed the expression of *edn* and *ednr* transcripts at stages 15, 17, and 21–28 via *in situ* hybridizations. This developmental series extends from mid-neurulation until the initial differentiation of the head skeleton. The expression of *L. japonicum ednA*, *ednC*, *ednE*, and *ednra* was previously described at mid-pharyngula stages^39^. While the expression patterns of these genes in these two lamprey species are generally similar, our analysis revealed additional expression domains not apparent in *L. japonicum*. These are detailed below.

#### Expression of ednA-F

**Figure 1:**
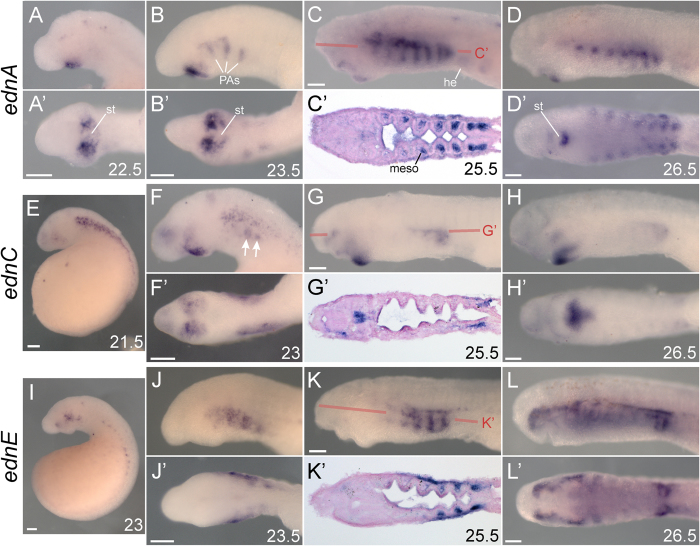
Expression summary of *edn*s in *P. marinus*. Left lateral views in all non-prime panels. All prime lettered panels show ventral/oral views of the correspondingly lettered panel, save C’, G’, and K’ which show horizontal sections. Developmental stage^68^ is indicated for each specimen in the bottom right of a panel. (**A–D**) *ednA* expression in *P. marinus* surrounds the stomodeum and marks the pharyngeal arches. (**E–H**) *ednC* expression in *P. marinus* is found around the stomodeum and on the lateral sides of the head. Arrows in F point to mesenchymal *ednC* expression below the superficial expression in the ectoderm. (**I–L**) *ednE* expression in *P. marinus* is found along the somites, and throughout the facial and pharyngeal skeleton. All scale bars represent 100 μm. he, heart; meso, mesoderm; PAs, pharyngeal arches; st, stomodeum.

*ednA* expression was first detected in ectoderm surrounding the forming mouth (stomodeum) weakly at st. 22, and more strongly at st. 22.5 (Fig. 1A). This expression domain was also found in *L. japonicum*^39^. This ectoderm is fated to eventually cover the nasohypophyseal plate, contributing to the epithelium of the nostril^47^. At st. 23.5, expression around the mouth appeared in a more distinct “comma” shape, and expression in the anteriormost dorsomedial pharyngeal arches (PAs) was first detected (Fig. 1B). Through st. 25.5, *ednA* expression in the pharynx expanded to the posteriormost PAs, while transcription became reduced around the mouth and in the anteriormost PAs (Fig. 1C). Sectioning at this stage revealed expression in both the center of the posteriormost seven PAs (in the mesodermal ‘core’, Fig. 1C’) as well as the ectoderm overlying these PAs. At st. 26.5, expression was reduced to a small patch of mesoderm within each of these PAs (Fig. 1D). This expression persisted until st. 27.5, also reappearing in the region of the dorsal PA1 (not shown). *ednB* expression was detected weakly in the upper lip, ear, heart, and somites at st. 25.5, and later in the brain of st. 28 larvae (Fig. S5A,B). *ednC* expression was first strongly detected at st. 21.5 along the flank in the ectoderm, and weakly in a medial spot within the anterior brain and in the ectoderm overlying lateral portions of the head (Fig. 1E). By st. 23, the ectodermal flank expression largely faded, while the brain expression expanded, and the expression flanking the head shifted more ventrally around the stomodeum to occupy a similar region as *ednA* transcripts (compare Fig. 1B’ to F’). Deeper mesenchymal expression in the posterior future PAs was also first detected at st. 23 underneath the faded flank expression (arrows in Fig. 1F). At st. 25.5, expression around the mouth shifted to mesenchyme of the lower lip, but still remained in tissues on the lateral sides of the mouth (Fig. 1G); only this lower lip expression was previously characterized in *L. japonicum*^39^. Sectioning revealed that all expression at st. 25.5 was mesenchymal (Fig. 1G’). At stages 26.5 (Fig. 1H) and 27.5 (not shown), the posterior PA expression was progressively diminished, and the more anterior expression around the mouth and in PA1 became progressively refined. *ednD* expression was not detected at any embryonic or larval stage assayed here. *ednE* transcripts were first visualized weakly at st. 22 in head ectoderm (not shown), and more robustly in the st. 23 head ectoderm and along the boundary between the anterior yolk and the somites (Fig. 1I). Up to st. 25.5, this superficial PA expression in the head proceeded in an anterior to posterior fashion, and was only present in the most posterior PA by st. 28 (Fig. 1J-L; st. 28 not shown). At st. 25.5 expression began in deeper mesenchymal tissues within the PAs (Fig. 1K’), and in a horizontal stripe dorsal to the PAs, presaging pigment deposition (Fig. 1K); this latter expression domain was previously described in *L. japonicum*^39^. *ednF* expression was detected at st. 25.5 diffusely throughout the entire head, but by st. 28 was mainly expressed in the brain (Fig. S5C–F).

#### Expression of P. marinus ednra and ednrb

**Figure 2:**
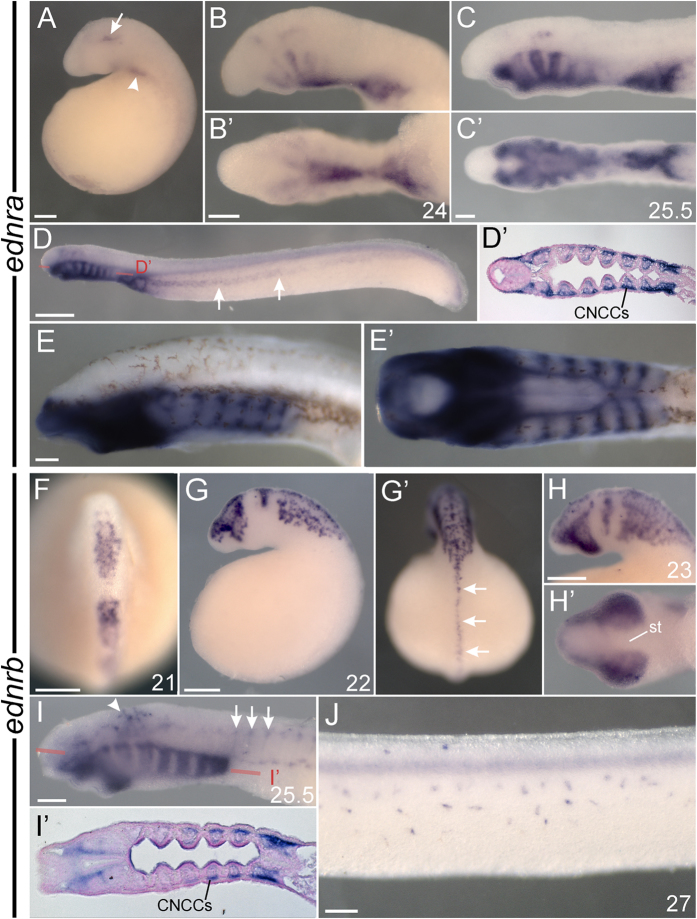
Expression summary of *ednr*s in *P. marinus*. Left lateral views in all non-prime panels save F, which is a dorsal view with anterior to the top. All prime lettered panels show a ventral view of the correspondingly lettered panel save D’ and I’, which are horizontal sections, and G’, which is a dorsal view with anterior facing away from the page. Developmental stage^68^ is indicated for each specimen in the bottom right of a panel. (**A–E**) *ednra* expression in *P. marinus* is found in mesoderm, the heart, and the post-migratory future head skeleton. Arrow in A indicates anterior mesoderm expression of *ednra.* Arrowhead in A indicates *ednra* expression in the nascent heart. Arrows in D indicate *ednra* expression consistent with lateral plate mesoderm. Red line labeled D’ in D indicates the approximate plane of section in D’. Note expression in the future head skeleton (CNCCs) shown in D’. (**F,G**) *ednrb* expression in *P. marinus* marks migratory NCCs. Arrows in I indicate expression consistent with dorsal root ganglia. Arrowhead in I indicates expression consistent with the earliest migrating pigment cells. Red line labeled I’ in I indicates the approximate plane of section in I’. Note expression in the future head skeleton (CNCCs) shown in I’. Arrows in G’ indicate trunk NCCs still poised at the neural crest. All scale bars represent 100 μm, save the scale bar in panel D which is 500 μm. NCCs, neural crest cells; st, stomodeum.

*P. marinus ednra* was first detected in a small paired patches of anterior mesoderm at st. 21 (Fig. S5G,H), and more robustly at st. 22 (Fig. 2A). At this stage, expression also began at the base of the pharynx where the heart will eventually develop (arrowhead in Fig. 2A). These expression domains were maintained until st. 27. ISH revealed *ednra* expression in late migratory and post-migratory skeletogenic NCCs in the head beginning at st. 24, first staining the ventralmost NCCs (future mucocartilage), as well as the upper and lower lips, and the more anterior PAs (Fig. 2B). PA expression thereafter proceeded to initialize in an anterior to posterior wave (Fig. 2B–D). By st. 26.5, this NCC expression was found throughout all PAs (Fig. 2D,D’). At st. 26.5 *ednra* was also detected in nascent lateral plate mesoderm (arrows in Fig. 2D), similar to expression of lamprey *Lbx-A*^48^. *ednra* transcription was maintained throughout st. 29, strongly marking the future head skeleton as cartilage began to differentiate (Fig. 2E). Most of these expression domains have also been observed in the Japanese lamprey^39^. *ednrb* expression was first detected at st. 21 in pre- and early migratory NCCs (Fig. 2F). By st. 22, these *ednrb*-positive cells in the head were migrating ventrally towards their destinations in the pharynx (Fig. 2G), while the majority of those in the trunk were still poised at the dorsal neural tube (arrows in Fig. 2G’). This migratory NCC expression continued through st. 24, becoming progressively extended ventrally and more diffuse as more NCCs had begun their migration (st. 23 shown in Fig. 2H). *ednrb* transcripts at st. 25.5 were detected around the mouth and in pre-skeletal NCCs in the PAs (Fig. 2I,I’), as well as future pigment cells (arrowhead in I) and peripheral nervous system components scattered along the lateral sides of the head, anterior yolk, and somites, including those that resemble dorsal root ganglia (arrows in Fig. 2I). At st. 26.5 expression in the pharynx was largely lost, though the expression in pigment cells was still apparent (not shown). Bleached larvae revealed this expression persists in melanophores until at least st. 28, being found in cells on the dorsal ridge and flank of the animals (st. 27 shown in Fig. 2J).

### Expression of *X. laevis* endothelin signaling components

We performed ISH for all *edn* and *ednr* genes spanning st. 17 to st. 40, (mid-neurulation to the onset of head skeleton differentiation). Except in the cases of *edn2* and *ednra*, all expressed *X. laevis*-specific gene duplicates analyzed here were discovered in completely overlapping domains at all stages assayed. We failed to clone the *X. laevis* duplicate *ednrb2-b*.

#### Expression of X. laevis edn1-3

**Figure 3:**
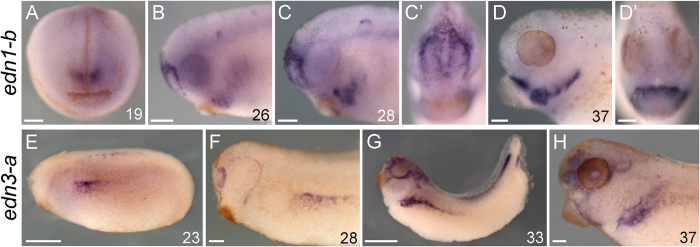
Expression summary of *edn*s in *X. laevis.* Left lateral views in all non-prime panels save (**A**), which is an anterior view. Prime panels show anterior views of the correspondingly lettered panel. Developmental stage^69^ for each specimen is indicated in the bottom right corner of a panel. (**A–D**) *edn1-b* expression in *X. laevis* marks ectoderm around the nasal placodes, and ventral non-NCC mesenchyme and epithelia in the pharyngeal arches. (**E–H**) *edn3-a* expression in *X. laevis* marks ectoderm where future *ednrb2-*positive cells will migrate. All scale bars represent 100 μm, save those in E and G which represent 500 μm.

*X. laevis edn1* was first detected at st. 19 in two patches of ectoderm just above the future stomodeum, overlying the developing telencephalon (Fig. 3A; see Fig. S6A–C for st. 28 sections). This expression persisted until st. 33, eventually becoming restricted to the ectoderm surrounding each nasal placode. At st. 26 we first observed *edn1* transcription within the ventral PAs, beginning in the region of the ventral hyoid stream. At later stages, expression of *edn1* spread posteriorly, then anteriorly to the rest of the ventral PA endoderm, mesoderm, and ectoderm, but not NCC-derived mesenchyme (Figs 3B–D and S6D–F). By st. 37 *edn1* was detected ventrally in each PA (Fig. 3D). To assay the expression of *edn2-a*, first a probe corresponding to 387 nt of coding sequence was amplified from cDNA (“cds probe”). This probe produced signal in many different tissues in the head, though this was found to be largely background staining in the brain cavities and in the ear, and possibly also in the notochord (Fig. S6H,I). Sectioning revealed that mesenchymal staining within the eye and surrounding the eye at st. 35 and 37 was within cells. We were unable to clone a fragment of the identified *edn2-b* coding region out of embryonic cDNA. Thereafter, riboprobes were designed mainly against a portion of the 3′ UTR of each *edn2-a* (645 nt) and *edn2-b* (684 nt), also including the last ~70 bases of coding sequence (“3′ UTR probe”). These were amplified from genomic DNA. Using these UTR probes, *edn2-a*, but not *edn2-b* was detected within the future pronephros from st. 30–37 (Fig. S6G), subsequently in dorsal pharyngeal mesenchyme surrounding the eye from st. 35–37, and within the eye. Interestingly, our *edn2-a* 5′ UTR and cds probes produced different staining patterns, despite having been designed to detect the same transcript. This may reflect differences in splice variants of the *edn2-a* gene. *edn2-b* transcripts were never detected. *edn3* expression was first seen at st. 23 surrounding in the nasal placodes, along the flank, and in the anterior trunk of the neural tube (Fig. 3E). Expression along the flank expanded and was maintained in lateral plate mesoderm surrounding the pronephros through st. 37, and ceased thereafter (Figs 3E–H and S6K). ISH signal around the dorsal neural tube proceeded in an anterior to posterior fashion, and was no longer detected by st. 37. In the head, expression in the nasal placode expanded and was joined by other expression in mesenchyme overlying the border between PAs 1 and 2 and around the eye (Fig. 3G,H).

#### Expression of X. laevis endothelin receptors

**Figure 4:**
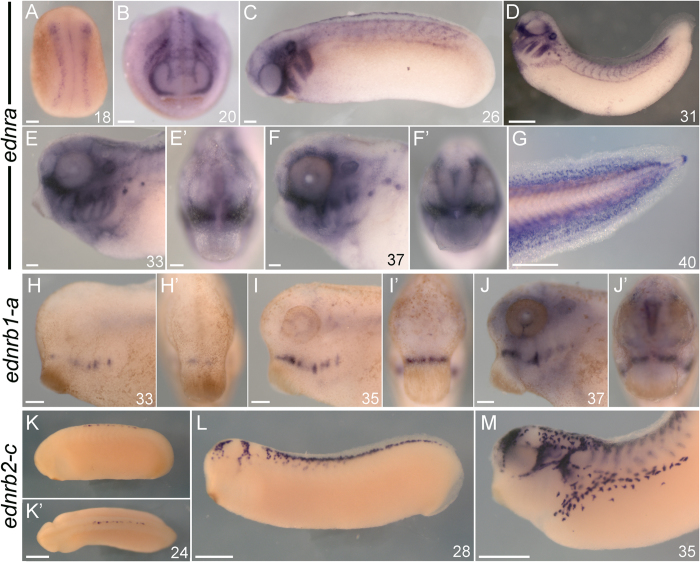
Expression summary of *ednr*s in *X. laevis.* Left-lateral views in all non-prime panels save (**A,B**), which are dorsal (**A**) an anterior (**B**) views. All prime panels show anterior/oral views of the correspondingly lettered panel save K’, which is a dorsal view. Developmental stage^69^ for each specimen is indicated in the bottom right corner of a panel. (**A–G**) *ednra* expression in *X. laevis.* (**A**) *ednra-a* uniquely marks pre-migratory NCCs. (**B–G**) *ednra-b* expression in migratory and post-migratory NCC derivatives, nephrostomes, the heart, and fin mesenchyme. (**H–J**) *ednrb1-a* expression in *X. laevis* marks the eye and medial pharyngeal arch NCCs. (**K–M**) *ednrb2-c* expression in *X. laevis* marks migratory pigment cells, nerves, and the forebrain. All scale bars represent 100 μm, save those in (**D,G,**K’**,L,M**) which represent 500 μm.

As previously described, *ednra-a* but not *ednra-b* expression was detected along the neural plate border during mid-neurulation (Fig. 4A). Thereafter, transcripts from both *ednra-a* (not shown) and *ednra-b* (Fig. 4B) genes were detected in completely overlapping regions. Expression was found in the otic placodes, uniformly throughout migratory cranial neural crest at st. 23, and along the flank in migratory trunk NCCs. These trunk NCCs could later be seen positioned between the boundaries of the somites, and aggregating along the future lateral line (Fig. 4C). By st. 31, expression had begun in the heart region, which continued throughout the entire series assayed here (Fig. 4C–E; st. 38–40 not shown). At stages 33–37, transcripts in the PA NCCs were apparently depleted ventrally compared to dorsal mesenchyme (Fig. 4D,E). Transcripts were also detected strongly in the medial fin at stage 40 (Fig. 4G). *ednra* transcripts were also detected in nephrostomes at stages 30–40 (Fig. 4D–F; arrows in Fig. S6R,S). *ednrb1* expression was observed only in the medial PAs from st. 31–40, and in the eye from st. 35–40 (Figs 4H–J and S6L–N; st. 38–40 not shown). *ednrb2-a* and *ednrb2-c* were both detected in pre-migratory and migratory nascent pigment cells, cranial ganglia, the forebrain, and peripheral nervous system derivatives of the trunk (Fig. 4K–M and S6O-Q).

## Discussion

### Duplication and specialization of Endothelin signaling pathways occurred in stem vertebrates

To better understand the ancestral roles of the endothelin pathway in vertebrate development, we performed an exhaustive search of lamprey genomic and transcriptome sequences for *edn* and *ednr* homologs. We then determined their expression throughout early development. To facilitate comparisons with gnathostomes, we also characterized and analyzed the embryonic and larval expression of all *edn*s and *ednr*s in *X. laevis*. To our knowledge, this is the first report detailing the embryonic and larval expression of all *edn*s and *ednr*s in a single gnathostome.

Phylogenetic analysis lends weak support to a scenario wherein a single ancestral *ednr* gene was duplicated in stem vertebrates, with *ednrb* later duplicated in stem gnathostomes (Fig. S3). Our data showing similar expression of lamprey and gnathostome *ednra* and *ednrb* paralogs supports this, with *ednra*s marking the heart and post-migratory skeletal NCCs, and *ednrb*s marking migrating pigment cells, cranial glia, and other peripheral nervous system precursors. Overall, duplication and functional specialization of *ednr*s is similar to that of other regulators of NCC development, including SoxE^49^, Tfap2^50^, Dlx^41^, and Id^51^,^52^, which all have multiple paralogs expressed in NCC. Furthermore, like SoxE^53^, Tfap2^50^,^54^, Id^51^,^52^, and FoxD^55^ there is evidence that duplication of *ednr*s was accompanied by biochemical subfunctionalization and/or neofunctionalization. In mouse, Ednra binds most strongly to Edn1 and Edn2, while mouse Ednrb (a type B1 receptor) binds all Edns with similar affinity^27^; in *X. laevis* Ednrb2 preferentially binds Edn3^28^. Furthermore, rescue experiments have shown that mouse Ednrb cannot rescue the function of Ednra in maxillary skeletogenic cranial NCC, due to the inability of Ednrb to signal through Gα_q_/Gα_11_ proteins^36^. Whether these differences reflect the loss of Ednrb functionality or a gain of Ednra functionality awaits testing of amphioxus Ednr, the only invertebrate Ednr known, and the only appropriate outgroup.

It is unknown if lamprey Ednra and Ednrb have diverged with regard to ligand binding affinities or signal transduction outputs. However, their divergent expression patterns support the hypothesis that distinct Ednra and Ednrb signaling pathways evolved in stem vertebrates. Because of the unique roles different Ednrs play in NCC migration and fate determination, it is tempting to speculate that duplication and divergence of vertebrate *ednr*s facilitated the evolution of the highly patterned and multipotent NCC of modern jawed and jawless vertebrates.

Given that Endothelin cleaving enzymes are responsible for the final processing of endothelin ligands, their appearance was a critical evolutionary step in the advent of the first vertebrate-type Endothelin pathway. In an attempt to understand when the first ECE appeared, we analyzed M13 metallopeptidases from a variety of deuterostomes. Given the available genomic and transcriptomic resources, we were unable to identify coding sequences most closely resembling ECE-1/2 in any invertebrate. However we did find coding sequences resembling the closely related ECE-like1 and Neprilysin in amphioxus (*Branchiostoma floridae*) and sea urchin (*Strongylocentrotus purpuratus*), and PHEX in sea urchin only. It seems probable then that the gene duplications giving rise this suite of genes (*ECE-like1*, *Nep*, and *PHEX*) predate the divergence of echinoderms and chordates. Given that recombinant *in vitro* experiments have shown that ECE-like1 is unable to cleave Big Endothelin 1^56^, and Neprilysin degrades Big Endothelin 1 in a manner that yields no detectable Edn1 ligand^57^, it seems plausible that the invertebrate genes identified here do not have a role in any type of rudimentary Endothelin ligand processing. Thus, in combination with the apparent absence of any *edn*-like gene outside of vertebrates^21^, there is no evidence that true Endothelin ligands or Endothelin converting enzymes exist outside of vertebrates based on deposited deuterostome sequences.

Despite the overall sequence similarity of amphioxus Ednr-like to vertebrate Ednrs, this invertebrate Ednr exhibits many divergent features, including the absence of one highly conserved lysine residue within the 2^nd^ transmembrane domain known to be important for ligand binding^20^,^58^,^59^. While it is possible that invertebrate chordate *edn*s or *ECE*s may have been overlooked during genome sequencing or annotation, the available data strongly suggest that early vertebrates greatly expanded on the elaborate biochemistry underlying these pathways. If no modern invertebrate ECE or Edn ligand truly exists, this would suggest that the Endothelin signaling pathway itself is a vertebrate synapomorphy, which thereafter diversified into a complex system of different ligands, processing enzymes, and receptors before the divergence of cyclostomes and gnathostomes.

### Differences in *ednr* expression reveals flexibility in the timing and extent of *ednr* transcription in NCC

While *P. marinus* and gnathostomes express their *ednra* and *ednrb* paralogs similarly, there are some clear differences (see Table 1). For one, lamprey *ednra* is restricted to NCCs destined for the head, while zebrafish^60^, *X. laevis*, chicken^61^, and possibly mouse^17^ all express *ednra* in trunk NCCs. Furthermore, while zebrafish^60^, Xenopus, and mouse^17^,^34^ express *ednra* in some migrating NCCs, lamprey and chicken^61^ do not express *ednra* in NCCs until they have reached their destinations. Whether these differences reflect an expansion of the ancestral *ednra* expression pattern in gnathostomes, or a restriction of *ednra* to post-migratory cranial NCC in lamprey and chicken is unclear.

*P. marinus* expresses *ednrb* more broadly in NCCs than any gnathostome, with strong expression apparent in the peripheral nervous system, pigment cells, and all skeletogenic NCCs during and briefly after migration. By contrast, Zebrafish^62^,^63^ and mouse^16^,^37^ restrict *ednrb*-type expression to pigment cells and peripheral nervous system derivatives, though early zebrafish *ednrbb* expression has not been well-described, leaving open the possibility of more widespread *ednrb* expression in this species. *X. laevis* expresses *ednrb*-type receptors in pigment cells, peripheral nervous system derivatives, and a minor subset of post-migratory skeletogenic NCCs. As in lamprey, avian *ednrb1*^15^,^64^ appears to broadly mark early migratory cranial NCCs, although it is unclear if this expression persists after those cells reach the pharynx. Based on these comparisons, we speculate that lamprey-like *ednrb* expression in all, or most, migrating NCC represents the ancestral vertebrate state, with the loss of some expression domains occurring after duplication of *ednrb* in the gnathostome lineage.

### *ednrb* duplicates in *X. laevis* highlight divergent subfunctionalization in different gnathostome lineages

Recent phylogenetic analysis suggests that *ednrb* was duplicated in stem gnathostomes, giving rise to *ednrb1* and *ednrb2*^20^. Most modern gnathostomes retain both duplicates, with the exception of zebrafish and therian mammals, which have both lost *ednrb2*^20^. In *X. laevis, ednrb2* is expressed in pigment cells, while *ednrb1* is only expressed in the eye, and a small population of post-migratory NCCs in the intermediate domain of the PAs, reminiscent of *dlx4*^42^. In quail and chicken embryos, *ednrb2* is also expressed in pigment cells. However, unlike *X. laevis*, both avian *ednrb* receptors are expressed in dorsal root ganglia and Schwann cells, though at different times^64^,^65^. Interestingly, both mouse and zebrafish appear to have compensated for the loss of *ednrb2* by expressing *ednrb1* in NCC-derived pigment cells^16^,^37^,^62^,^63^, an expression domain not seen in any other gnathostome examined to date. The differential ligand binding properties of *ednrb1*^27^ and *ednrb2*^28^ open up the possibility that these divergent expression patterns may reflect lineage specific-differences in the migration patterns and/or fate of *ednrb*-expressing NCC, in particular, pigment cells.

### Endothelin receptor expression in pre-migratory neural crest supports an ancestral function in NCC specification

Available expression data shows that only *X. laevis* and *P. marinus* transcribe an *ednr* in pre-migratory NCC in a pattern reminiscent of neural crest specifiers such as *Sox9*/*SoxE2* and *FoxD3*/*FoxD-A*^11^,^66^. Work in *X. laevis* suggests a role for early Ednra signaling in the maintenance of NCC identity and regulation of neural crest specifiers^11^. In *P. marinus*, *ednrb* rather than *ednra* is expressed broadly in premigratory NCC (Fig. 2H), however it is unknown if it performs a similar function.

### Similar expression domains support shared function of some lamprey and gnathostome Endothelin ligands

**Figure 5:**
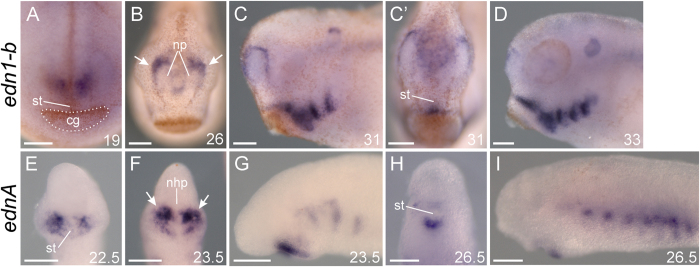
Lamprey *ednA* expression is reminiscent of gnathostome *edn1* expression, despite low support for strict orthology. Oral/anterior views in (**A–**C’**,E,F,H**). Left lateral views in (**C,D,G,I**). Developmental stage^68^,^69^ for each specimen is indicated in the bottom right corner of a panel. (**A–D**) *X. laevis* expression of *edn1-b* from stage 19 to 33. Dotted outline in A surrounds the cement gland. Arrows in B indicate expression in ectoderm which will eventually contribute to the nostrils (**E–I**) *P. marinus* expression of *ednA* from stage 22.5 to 26.5. Arrows in F indicate expression in ectoderm which will eventually move rostrally to cover the nasohypophyseal plate, contributing to the future medial nostril. In a similar sequence, both genes are expressed in bilateral patches above the future mouth, surrounding the nasal placodes/nasohypophyseal plate, in pharyngeal arch mesoderm and ectoderm, and just below the stomodeum. All scale bars represent 100 μm. cg, cement gland; nhp, nasohypophyseal plate; np nasal placode; st, stomodeum.

**Figure 6:**
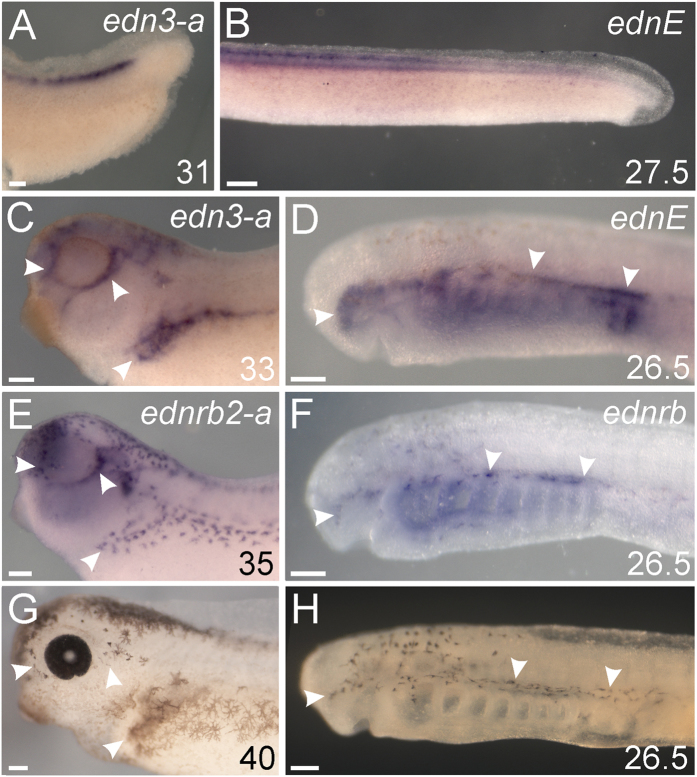
Lamprey *ednE* and gnathostome *edn3* expression patterns both predict neural crest-derived melanophore migration routes. All panels show left lateral views. Developmental stage^68^,^69^ for each specimen is indicated in the bottom right corner of each panel. ISH panels (**A-F**) are labeled with the targeted gene transcript in the top right corner. Arrowheads in C and D mark regions where *edn3-a* (**C**) or *ednE* (**D**) are expressed. Arrowheads in E and F indicate cells expressing *ednrb2-a* (**E**) and *ednrb* (**F**). Panels G and H show light microscopy photos of larval *X. laevis* (**G**) and *P. marinus* (**H**). Arrowheads in (**G,H**) indicate areas where pigment cells migrated. All scale bars represent 100 μm.

Despite poor sequence similarity, gene expression patterns lend support to shared functions of some lamprey and gnathostome Endothelin ligands. Specifically, gnathostome *edn1* and lamprey *ednA* have similar expression patterns around the future mouth, pharynx, and in the presumptive nasal epithelia^47^ (Fig. 5; arrows in Fig. 5B,F), while gnathostome *edn3* and lamprey *ednE* have broadly similar expression in domains populated by *ednrb*-expressing melanophores (Fig. 6). While these similarities may be due to the direct orthology of these genes, it could also be due to similar regulatory subfunctionalization of non-orthologous Edns produced by duplications before or after the gnathostome/agnathan split. Such convergent subfunctionalization could have been facilitated by the organization of the ancestral Edn cis-regulatory landscape.

Phylogenetic analysis suggests a fourth Endothelin ligand gene, *edn4*, was lost in lobe-finned fish; its embryonic expression pattern has not been described. It is possible one of the other lamprey *edn*s is an *edn4* ortholog. Embryonic *edn2* expression is only known in *X. laevis* (this work), and does not strongly resemble any of the lamprey *edn*s alone (save the dorsal pharyngeal expression found in most lamprey *edn*s). Further analyses of *edn2* and *edn4* may resolve these apparent discrepancies.

### Differences in *edn1/ednA* expression correlate with differences in gnathostome and agnathan head skeleton patterning

While *edn1 and ednA* have broadly similar expression patterns, there are some clear differences. In gnathostomes, *edn1* is expressed in the ectoderm, mesoderm, and endoderm of the ventralmost pharynx, where it acts through Ednra to drive expression of the ventral specifier genes *hand1* and *dlx5/6*^9^,^14^,^34^,^35^,^60^. In lamprey, *ednA* transcripts are restricted to the pharyngeal mesoderm and ectoderm in the dorsal-intermediate domain of the pharynx. Unlike gnathostomes, this expression does not abut *hand*-expressing NCC in ventral pharynx^41^ (Fig. 1). The significance of this positional difference in lamprey *ednA* and gnathostome *edn1* is unclear, but we speculate that it may relate to differences in the dorso-ventral patterning of the pharyngeal skeleton between these groups, namely the pronounced asymmetry in gnathostome PAs as compared to lamprey.

## Conclusions

To our knowledge, lamprey has the most *edn* ligands expressed during early development of any vertebrate. This could be the result of cyclostome- or lamprey-specific duplications, and/or the loss of *edn* ligand genes in gnathostomes. Regardless, the presence of multiple *edn*s and *ednr*s in *P. marinus* strongly suggests that sophisticated Endothelin signaling in NCCs predates the divergence of modern jawed and jawless vertebrates. Given the essential involvement of these pathways in NCC guidance and differentiation, we posit that Endothelin receptor duplication and sub- and/or neofunctionalization played a role in the evolution of the multipotent and highly patterned NCC of modern vertebrates. New methods for high-efficiency mutagenesis^67^ in lamprey should allow us to better deduce the ancestral roles of Edn signaling in vertebrates and test this hypothesis.

## Additional Information

**How to cite this article**: Square, T. *et al*. Embryonic expression of *endothelins* and their receptors in lamprey and frog reveals stem vertebrate origins of complex Endothelin signaling. *Sci. Rep.* **6**, 34282; doi: 10.1038/srep34282 (2016).

## Supplementary Material

Supplementary Information

Supplementary Table S1

Supplementary Table S2

## Figures and Tables

**Table 1:** A summary of *ednr* subfunctionalization in vertebrates.

	Migratory, pre-skeletal NCCs	Post-migratory, Pre-skeletal NCCs	Migratory trunk NCCs (non-melanophores)	Post-migratory DRGs	Heart (post-migratory NCCs?)	Migratory and post-migratory melanophores
Sea lamprey	*ednrb*	*ednrb*, *ednra*	*ednrb*	*ednrb*	*ednra*	*ednrb*
Zebrafish	*ednra1*^60^	*ednra1* + *ednra2*^60^	*ednra1*^60^	*ednrb1*^62^,^63^	*ednra*^60^	*ednrb1*^62^,^63^
*Xenopus laevis*	*ednra*	*ednra* + *ednrb1* (minor subset)	*ednra*	*ednrb2*	*ednra*	*ednrb2*
Chick/quail	*ednrb1*^64^	*ednra*^61^	*ednrb1*^64^	*ednrb1*^64^ + *ednrb2*^65^	*ednra*^61^	*ednrb2*^65^
Mouse	*ednra*^17^,^34^	*ednra*^17^,^34^	*ednra* (?)^17^,^33^ + *ednrb1*^16^,^37^	*ednrb1*^16^,^37^	*ednra*^17^,^34^	*ednrb1*^16^,^37^

Note: Genes are separated by commas to indicate a temporal change, while a + sign indicates they are coexpressed. For *X. laevis,* “*ednra”* indicates that both the “*-a*” and “*-b*” copies are coexpressed. All zebrafish duplicates are listed by their specific name. Mouse *ednrb* is referred to here as *ednrb1* since it is an *ednrb1* gene (see Fig. S3). DRGs, dorsal root ganglia; NCCs, neural crest cells.
