# Supplementary material for: Embryonic expression of endothelins and their receptors in lamprey and frog reveals stem vertebrate origins of complex Endothelin signaling
Source: Sci Rep. 2016 Sep 28;6:34282. doi: 10.1038/srep34282 (PMC5039696; doi:10.1038/srep34282)
Supplement: Supplementary Information [file srep34282-s1.doc]

# Supplementary Figures for the MS:

# Embryonic expression of endothelins and their receptors in lamprey and frog reveals stem vertebrate origins of complex Endothelin signaling

Tyler Square, David Jandzik, Maria Cattell, Andrew Hansen, and Daniel Meulemans Medeiros


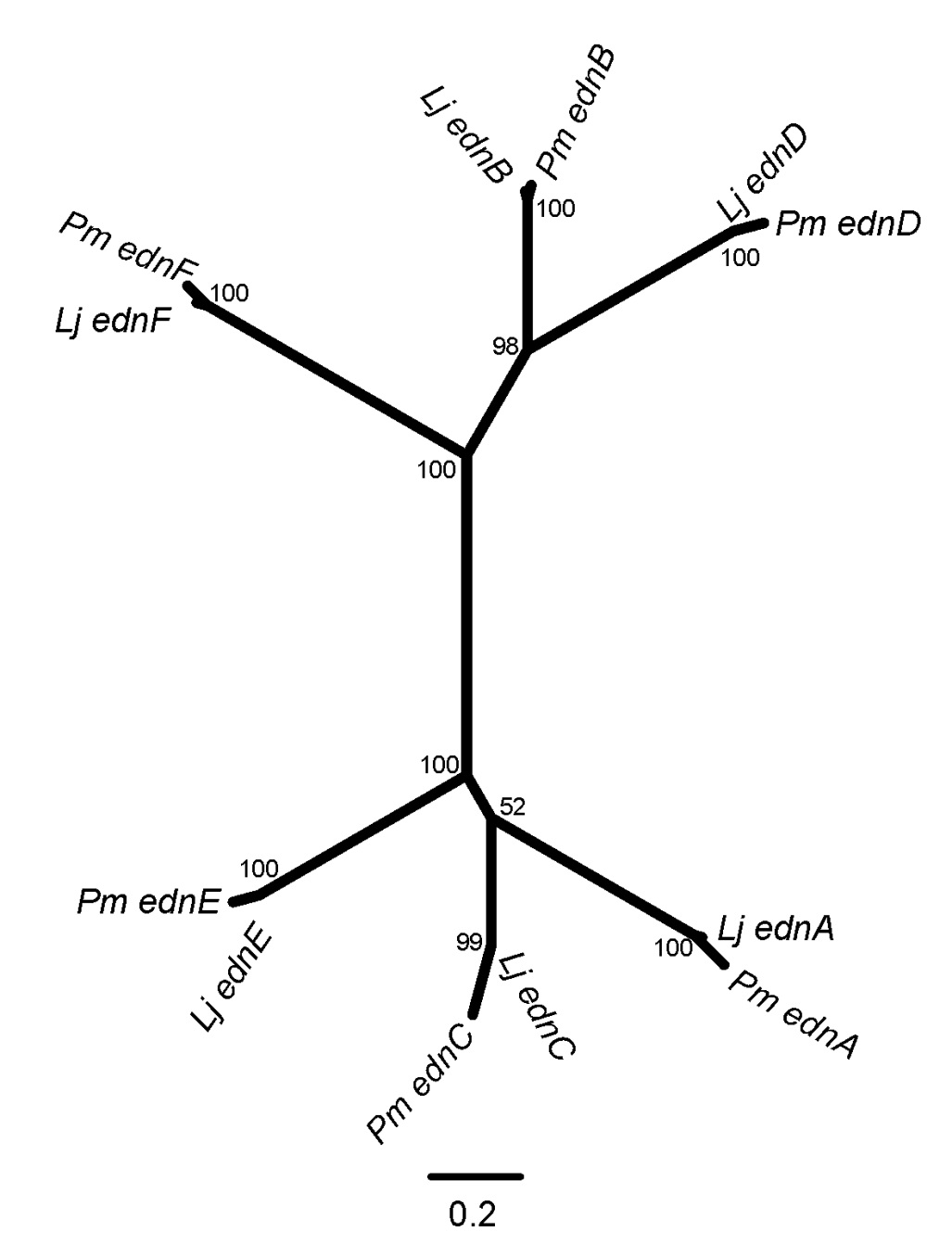


**Fig. S1**. An unrooted phylogenetic tree built from *edn* gene sequences in sea lamprey (*Petromyzon marinus*) and the Japanese lamprey (*Lethenteron japonicum*). Maximum likelihood analysis was used to determine lamprey ligand orthology. Bootstrap values are shown at the base of each node. Accession numbers for all sequences can be found in Tab. S2.


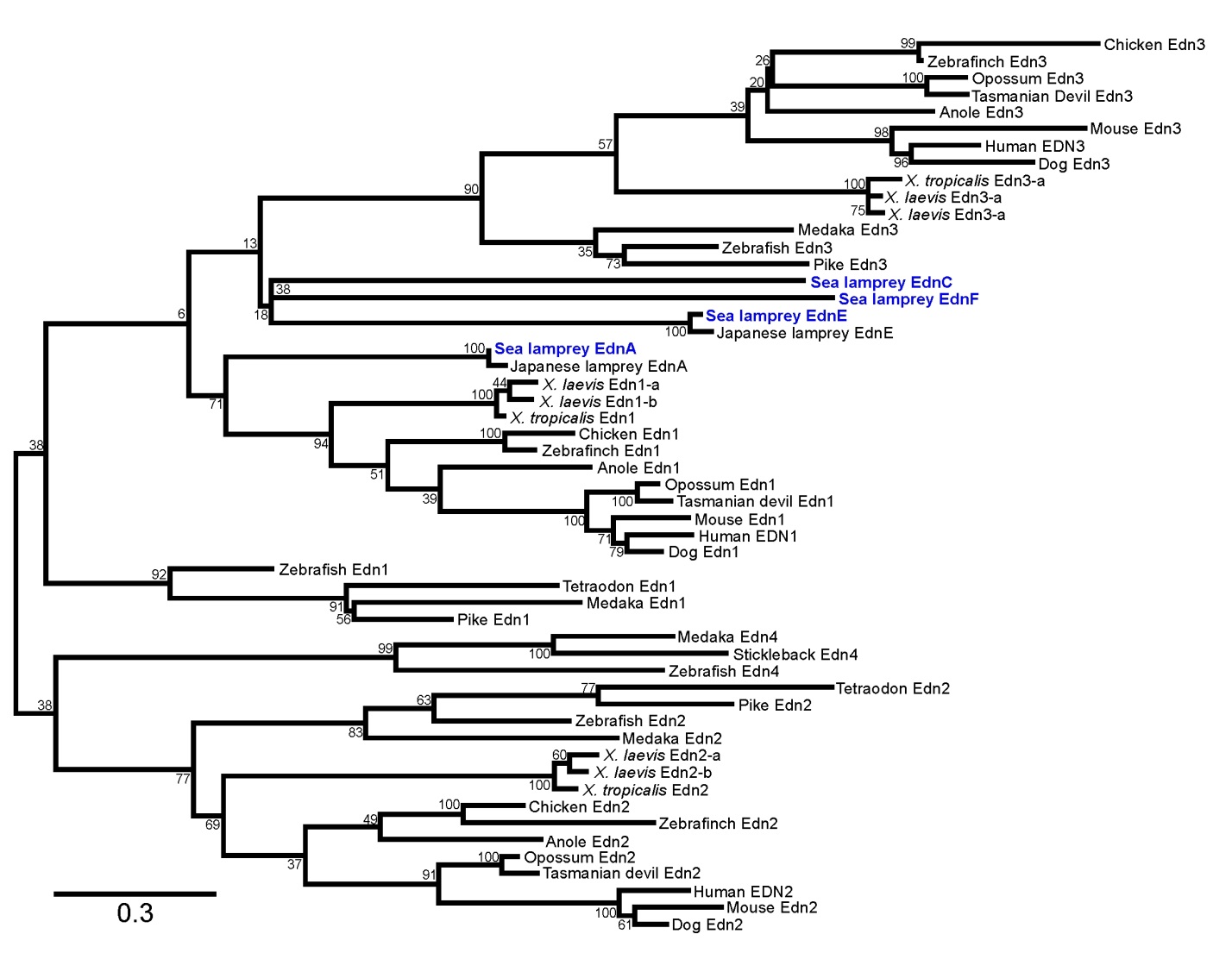


**Fig. S2**. A phylogenetic tree built from Edn amino acid sequences in vertebrates. Maximum likelihood analysis was used in attempt to determine the orthology of lamprey Edn ligands. This tree was midpoint rooted between the Edn1/3 clade and the Edn2/4 clade. All calculated bootstrap values are shown at the base of each node. Accession numbers for all sequences can be found in Tab. S2.


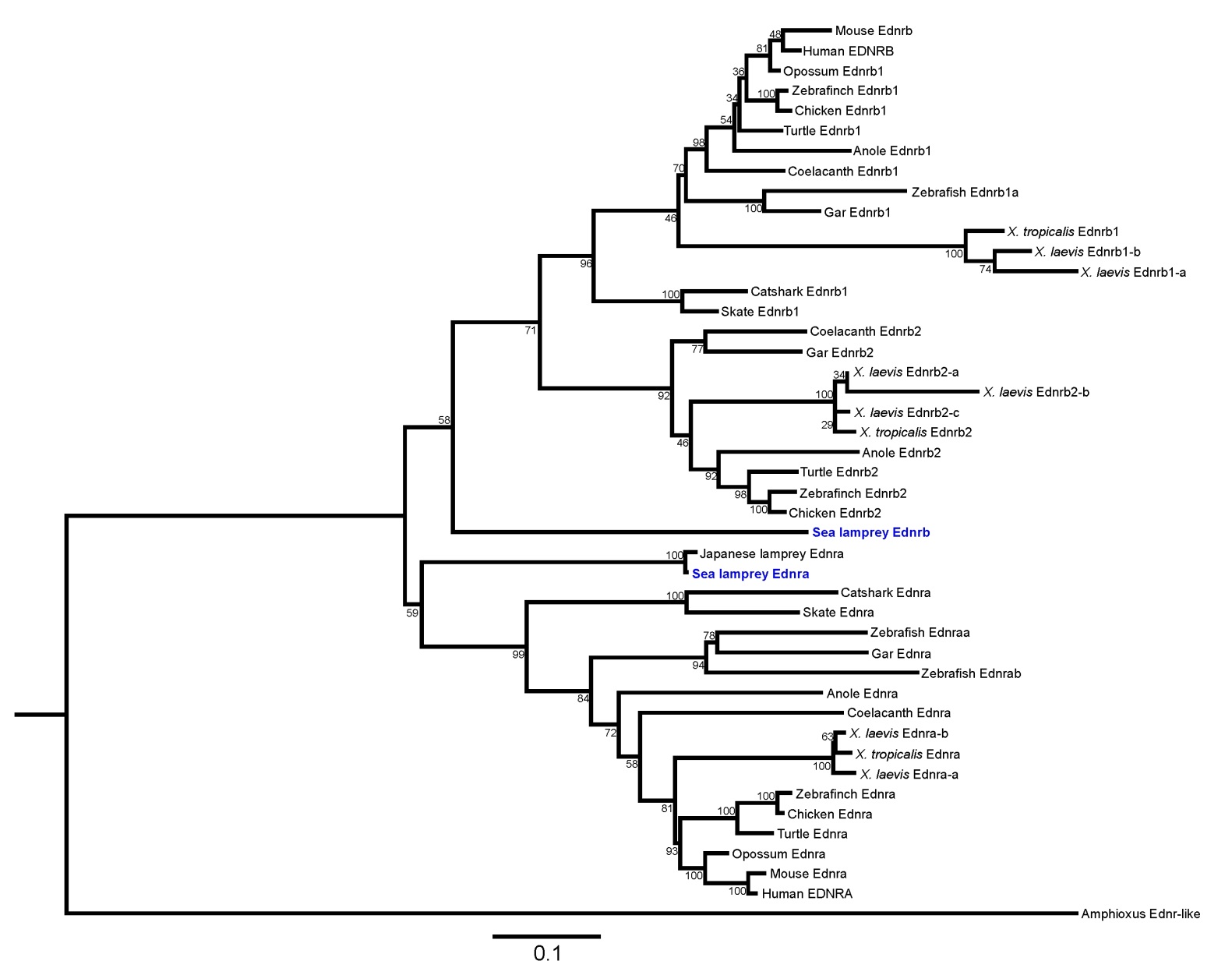


**Fig. S3**. A phylogenetic tree built from EdnR amino acid sequences in chordates. Maximum likelihood analysis was used to assign orthology to the lamprey EdnRs. All bootstrap values are shown at the base of each node. Amphioxus EdnR-like was selected as the outgroup. Accession numbers for all sequences can be found in Tab. S2.


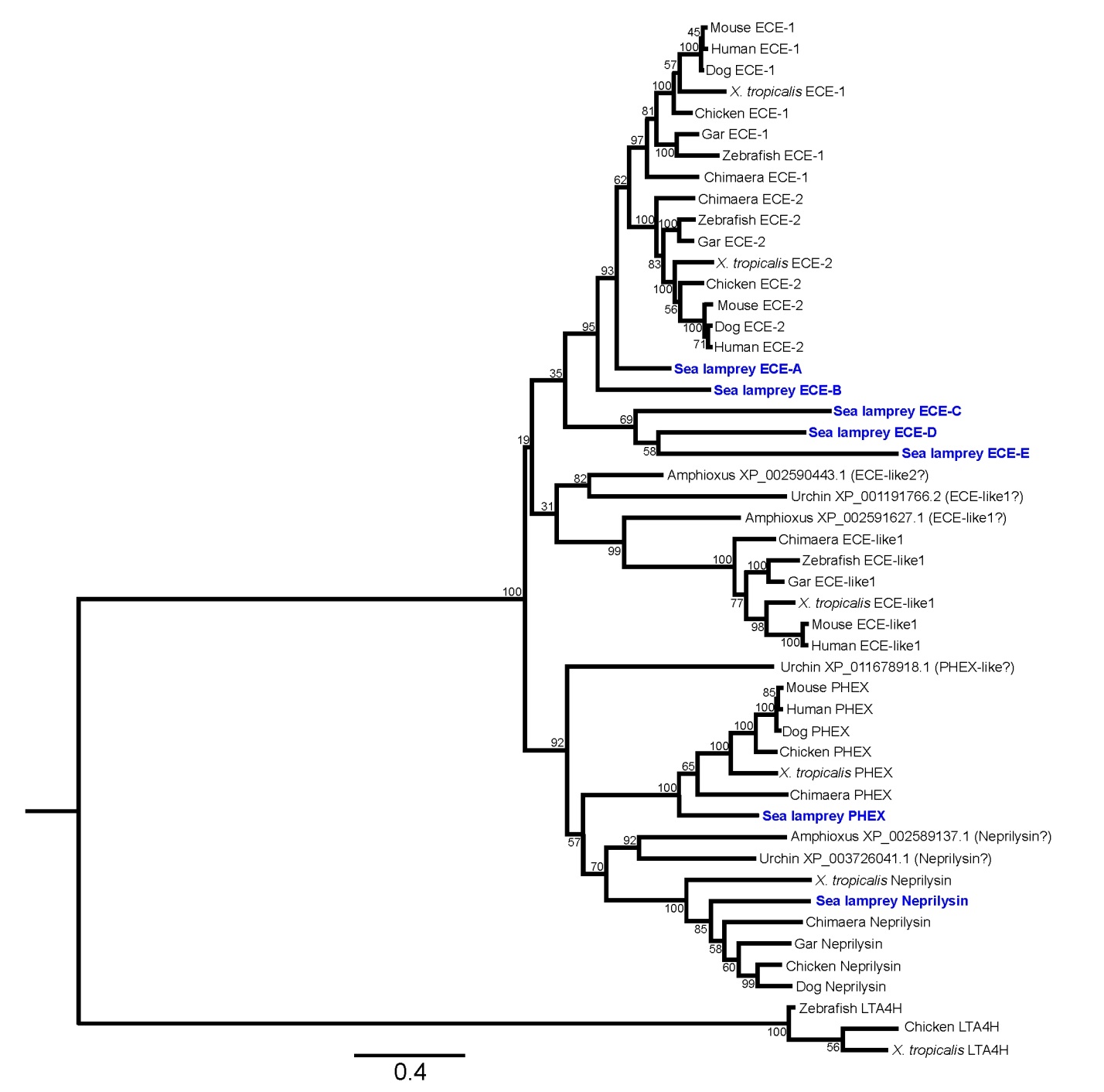


**Fig. S4.** A phylogenetic tree built from ECE-related metallopeptidase amino acid sequences in deuterostomes. Maximum likelihood analysis was used to assign orthology to the lamprey ECEs. All bootstrap values are shown at the base of each node. Three gnathostome LTA4H sequences were selected as the outgroup. Accession numbers for all sequences can be found in Tab. S2.


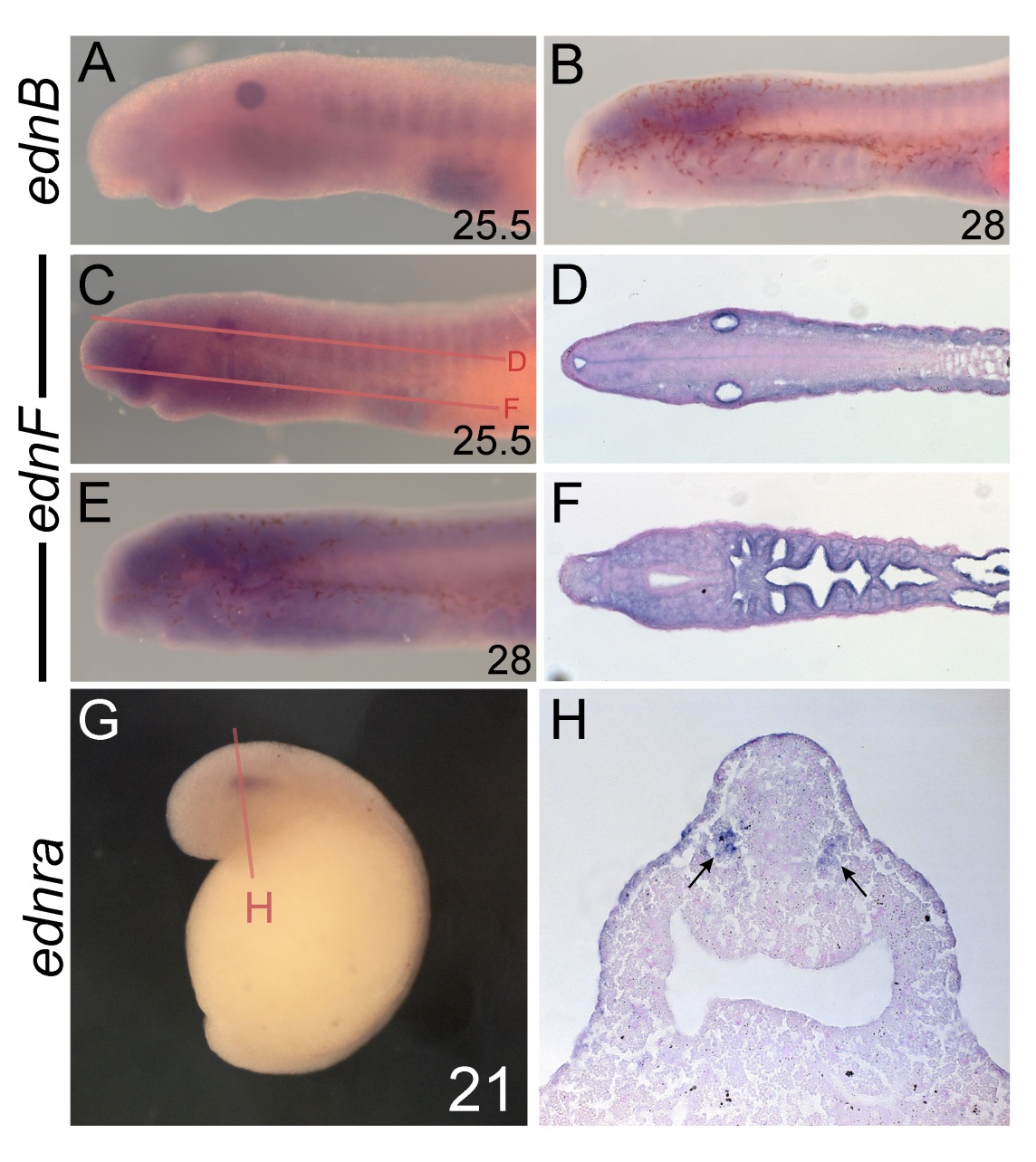


**Fig. S5**. Expression of *ednB, -F*, and *-ra* in *P. marinus* at pharyngula stages. Left lateral views in panels A, B, C, E, and G. Sections shown in panels D, F, and H correspond to the labeled red line in the wholemount ISH panel for each gene. Developmental stage^68^ is indicated in the bottom right corner of the wholemount panel for each specimen.


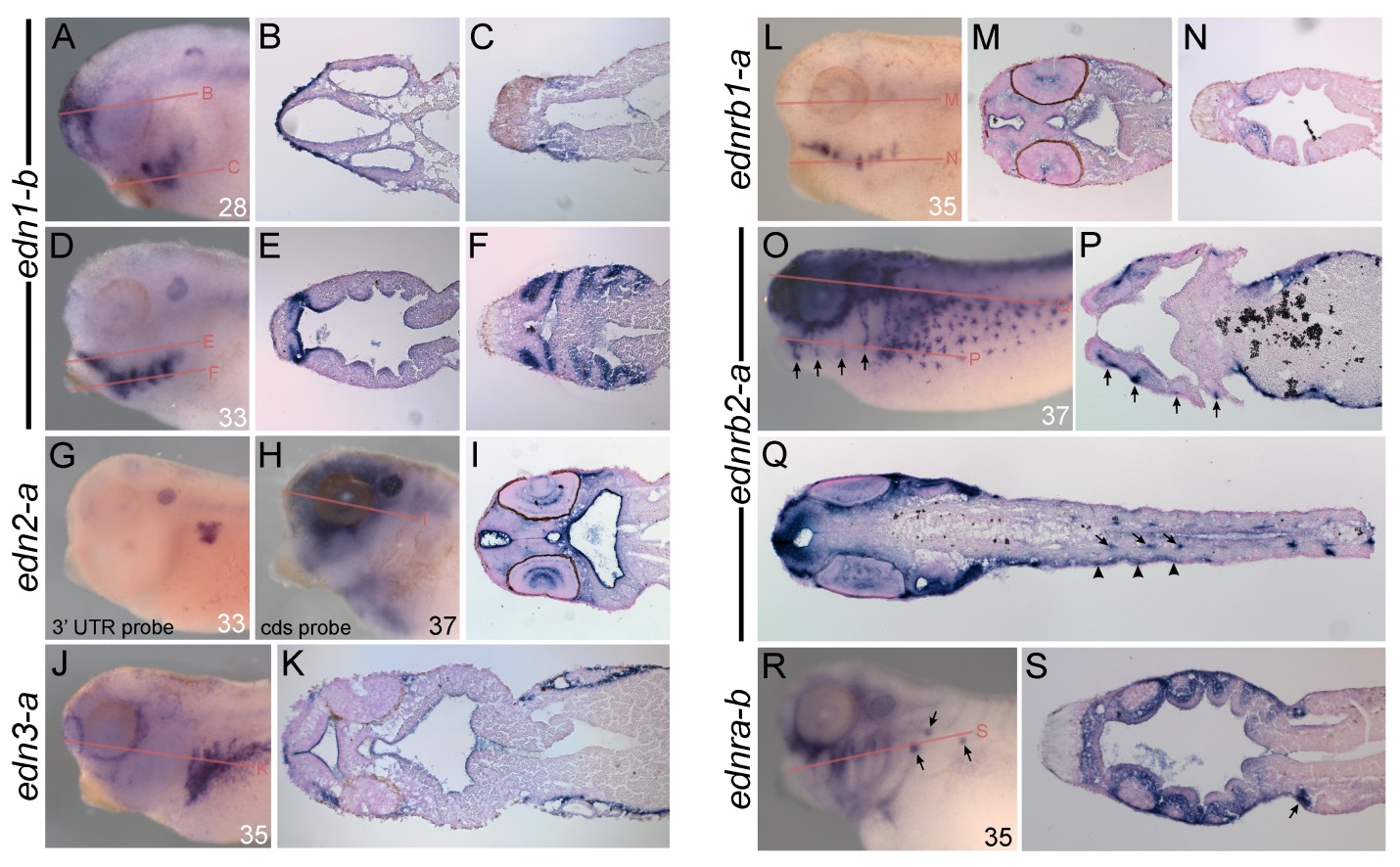


**Fig. S6**. Expression of *edn1-3*, *ednra*, and *ednrb* in *X. laevis.* Left lateral views in panels A, D, G, H, J, L, O, and R. Sections shown in panels B, C, E, F, I, K, M, N, P, Q, and S correspond to the labeled red line in the wholemount ISH panel for each gene. Developmental stage^69^ is indicated in the bottom right corner of the wholemount panel for each specimen. Note strong background staining with the *edn2-a* CDS probe in the brain (I). Arrows in O and P indicate *ednrb2-a* expression in the branchial nerves. Arrows in Q indicate expression consistent with dorsal root ganglia. Arrowheads in Q indicate expression in melanophores. Arrows in R and S indicate *ednra-b* expression in nephrostomes.
